# Rapporto sulla popolazione: Le molte facce della presenza straniera in Italia edited by Salvatore Strozza e Gustavo De Santis

**DOI:** 10.1186/s41118-018-0033-y

**Published:** 2018-04-24

**Authors:** Anthony C. Masi

**Affiliations:** Desautels Faculty of Management, McGill University, Montreal, Québec Canada

Bologna: Il Mulino, 2017, 200pp., ISBN: 978-88-15-26,798-6, Paperback, €16,00.

In 2017, at least ten reports or monographs were published in Italy by various public and private bodies on different aspects of immigration to the country, demonstrating that this topic is of considerable interest to scholars, policy-makers, and the general public. The study being reviewed here was issued under the auspices of the Italian Association for Population Studies of the Italian Statistical Society (AISP-SIS). It stands out as a particularly significant and indispensable contribution for those who want to understand the magnitude and the importance of the phenomenon of international immigration to Italy. The various essays in this volume examine in a broad, deep, and clear manner the “foreign presence” in Italy and implications of the latter for present and future developments. This report is the sixth promoted by the AISP-SIS on the demographic situation of the country. Two previous studies presented a general overview of Italy at the beginning of the twenty-first century (2012) and 150 years after the Unit (2014), while the other volumes of the series took into consideration specific aspects of the population: health and survival (2013), sexuality and reproduction (2015), and Italy in the economic crisis (2016).

The book’s value derives, in part, from the large number of researchers (from universities, the Italian National Statistics Institute, and policy-think tanks) who were contributors. The titles of the various chapters are (I) “International migrations and immigrant populations in Europe and in Italy”; (II) “Italy’s transition from country of emigration to country of immigration”; (III) “Demographic behaviours”; (IV) “Foreigners and the labour market”; and (V) “Aspects of integration”. To help readers better understand certain crucial points, most chapters contain “explanatory boxes” that provide further details and interpretations. The excellent editorial work of Strozzo and De Santis ensured stylistic continuity and coherence across the various chapters that were written by teams of authors. The result is an easy to read volume, rich in data and information, geared to help in understanding the topics in all their nuances, and containing explanations useful to address the ubiquitous “fake news” concerning Italy’s foreign population. This book traces the evolution of the number, distributions, and characteristics of foreigners in Italy, both legally resident and irregularly domiciled, over the last 10 years, and in some analyses over 20 or more years.

It is not possible here to mention all the data and information contained in this relatively brief but very thorough report. I will limit myself to mentioning some indicators that I found particularly salient. At the beginning of 2016, ISTAT estimated that there were just over 5 million immigrants in Italy out of a total population of almost 61 million, that is 1 out of 12 people in living country. Using official data and the classic tools of demography to project the trend of the Italian population from 2002 to 2015, the authors found that without foreign residents, the total population in Italy would have been only 57 million or 4 million less. In other words, the Italian population is not growing; indeed, it is not maintaining itself, except for migratory flows from abroad. The ageing processes of Italy’s population, already noted for some time, would be even faster without foreigners: the population over age 65 is currently 22%, but it would have been 23%. With many of the country’s welfare institutions based on the “pay as you go” system, that 1% less is important. In 2050, an Italy without foreigners could expect to have 37% of the population in the category of “seniors”, instead with foreigners that estimate drops to 33%—still high but contained to some extent. The report illustrates the importance of foreign presence in Italy not only for its weight in the demographic composition but also for its contribution to the country’s economy. As noted above, foreigners are around 8% of the total population, but they contribute 15% of the births (Italy does not recognise *ius soli*). Considering also that the average age of foreigners is much lower than that of Italians, these factors slow the ageing of the country’s population. In general, in the labour market, foreigners fill jobs that have been abandoned for some time by Italians. According to this report, fewer than 1 Italian in 12 has an unskilled job, while almost 5 out of 12 foreigners have this type of employment. Given the number of years of education that the foreign-born residents completed in their countries of origin, compared to Italians, they are *over-educated* for the type of work they do. Along these same lines, foreigners are more likely than native-born Italians to be *under-employed*.

The presence and characteristics of foreigners in Italy are not uniform throughout the country. There are a number of differences in migrant accommodation between the south and the rest of the country. Table 4.1 (page 103) compares the employment and unemployment rates between foreigners and the native-born Italian population, highlighting important, but also disconcerting, divergences among large geographical areas. In the north and centre of the country, when compared to immigrants, Italians have higher rates of employment and lower rates of unemployment. The situation is reversed in the south and in the Islands. There Italians have lower employment rates than foreigners, but higher unemployment.

Another important change highlighted in the report is that from 1996 to 2016, the countries of origin of the immigrants changed considerably (Tab. 2.3, p. 49). In 2016, former Yugoslavia, Tunisia, Germany, France, the United Kingdom, and Senegal are no longer among the top 10 countries of origin as they were in 1996. They were replaced by Romania, China, Ukraine, Moldova, India, and Bangladesh. The countries that remained in the top ten are Albania, Morocco, the Philippines, and Egypt, although their previous position in the ranking has changed.

The meticulousness of the analysts and the wealth of data presented create a complementarity between information and interpretation that is fundamental in negating some of the false notions regarding the heavy costs that immigrants are often presumed to impose on Italy. By controlling for age structure differences between immigrants and native-born Italians, and taking into account the “healthy migrant effect”, foreigners are much less likely to suffer from chronic diseases. This means that immigrants use fewer expensive health services. In fact, directly comparing costs (government expenditure) with benefits (the contribution of foreigners to GDP), the balance (contribution minus expenses) is clearly positive, and for 2015, it was estimated to be over 2.2 billion euros.

In the 2017 report, however, there are five important aspects that have not been sufficiently elaborated and which, in my opinion, deserve further investigation. The first three concern problems relating to the integration of foreigners into Italian society. First, statistics show that only around 3% of eligible immigrants are applying for Italian citizenship. Why so few? Second, foreign students at all levels, even in the second generation, have a lower academic performance than Italian students. Why is this the case? Third, various surveys indicate that only 1 in 2 of foreign students either born in Italy or who arrived before starting elementary school, feel fully “Italian”. What obstacles are preventing the rest from taking on this identity?

The other two aspects refer to changes in the motivations of foreigners’ requests to reside in Italy. In 2007, 3 out of 10 requests for long-term residence were for family reasons, while in 2015 these became almost 1 in 2. Italian immigration has evolved, in my opinion, in a way similar to what happened in the history of immigration to the Americas in past centuries. Movements of “sojourning” have morphed into movements of “settlement”, but this phenomenon occurred in Italy in a rather short time compared to the Americas. Are Italian institutions and society capable of adapting to such sudden changes and welcoming these new families? Finally, between 2007 and 2015 (Table 2.1, page 38), there was a considerable increase in the absolute number (from 9971 to 67,271) and in the percentage (from 3.7 to 28.2%) of applicants requesting asylum. Where is the problem—with the policy or with the implementation of the Italian system of “regular” immigration?

In conclusion, this report on the “many faces” of the foreign presence in Italy presents data, information, and interpretations of the experiences and behaviours of people born abroad and their children; offers suggestions for developing appropriate positions for Italian immigration policies; indicates the futility of continuing to distinguish between economic migrants and refugees; and stimulates reflection on recent decreases in migration flows to Italy. This book is an indispensable reference for discussions, debates, university courses, policy formulation, and political decisions regarding international immigration to Italy.

